# Epidemiological and Clinical Characteristics of Adult RSV Infections: A Retrospective Analysis at University Hospital Center Zagreb (2022–2024)

**DOI:** 10.3390/pathogens14030284

**Published:** 2025-03-14

**Authors:** Antonio Perčinić, Tara Vuletić, Nina Lizzul, Andrea Vukić Dugac, Ana Gverić Grginić, Irena Tabain, Dragan Jurić, Ana Budimir

**Affiliations:** 1 Department of Clinical Microbiology, Prevention, and Infection Control, University Hospital Center Zagreb, Kišpatićeva 12, 10000 Zagreb, Croatia; npredavec@gmail.com (N.L.); ana.budimir@kbc-zagreb.hr (A.B.); 2 Department of Respiratory Infections, University Hospital Center Zagreb, Kišpatićeva 12, 10000 Zagreb, Croatia; taravuletic123456@gmail.com (T.V.); andrea.vukic.dugac@kbc-zagreb.hr (A.V.D.); 3 Microbiological Service, Croatian Institute of Public Health, 10000 Zagreb, Croatia; ana.gveric-grginic@hzjz.hr (A.G.G.); irena.tabain@hzjz.hr (I.T.); dragan.juric@hzjz.hr (D.J.)

**Keywords:** respiratory syncytial virus (RSV), adult RSV infections, chronic diseases, RSV complications, RT-PCR

## Abstract

Respiratory syncytial virus (RSV) is a significant cause of respiratory infections in adults, particularly among older adults and individuals with chronic diseases. While traditionally linked to pediatric populations, RSV’s impact on adults, especially the elderly, is increasingly recognized but remains understudied in many regions. This retrospective study, conducted at the University Hospital Center Zagreb from October 2022 to April 2024, is the first to analyze RSV-positive adults in Croatia. Using RT-PCR testing, we evaluated clinical and epidemiological characteristics in both hospitalized and outpatient populations, focusing on those aged > 65 years. Among 2631 tested individuals, the RSV prevalence was 5.25%, with older adults experiencing the most severe outcomes, including pneumonia, COPD exacerbation, and intensive care admissions. Seasonal analysis confirmed a winter peak in RSV cases, while chronic conditions such as cardiovascular and respiratory diseases were strongly associated with higher complication rates. These findings demonstrate that older adults with comorbidities bear the greatest burden of RSV infection, highlighting the need for the early identification of high-risk patients. By providing detailed insights into RSV-related outcomes in this population, this study supports the development of targeted prevention and management strategies to reduce the burden of RSV in vulnerable groups.

## 1. Introduction

Respiratory syncytial virus (RSV) is a single-stranded RNA virus from the family Pneumoviridae and a major cause of respiratory tract infections worldwide. Although traditionally associated with pediatric populations, RSV is increasingly recognized as a significant pathogen in adults, particularly among older adults and individuals with chronic conditions or immunosuppression [1,2].

Globally, RSV is responsible for a substantial disease burden in adults. In the United States, RSV is estimated to cause between 60,000 and 160,000 hospitalizations and 6000 to 10,000 deaths annually among adults aged ≥ 65 years [3]. In the United Kingdom, RSV is estimated to cause approximately 8482 deaths annually, predominantly affecting individuals aged 65 years and older [4]. Another study analyzing global seasonal trends found that RSV activity peaks during the winter months in temperate regions, with a burden comparable to that of influenza [5]. Despite these findings, RSV remains underdiagnosed in adults due to clinical overlap with other respiratory infections, such as influenza and SARS-CoV-2, and the absence of routine RSV testing in many healthcare settings [6,7].

In adults, RSV infections range from mild upper respiratory tract symptoms, such as rhinorrhea and nasal congestion, to severe lower respiratory tract infections (LRTIs), including pneumonia and respiratory failure [6,7]. Chronic conditions such as cardiovascular and respiratory diseases, diabetes, and hematologic disorders significantly increase the risk of severe disease, complications, and hospitalization, particularly in older adults [2,8]. These factors, coupled with age-related immune decline, make older adults especially vulnerable to prolonged hospital stays and intensive care needs [7,9].

While RSV-related morbidity in adults is comparable to that of influenza, its public health impact remains underestimated due to lower awareness and the absence of established vaccination programs for older adults [9]. Although new RSV vaccines for older adults have been recently approved, their implementation remains limited, and their impact on disease severity is still under investigation [3]. Furthermore, the emergence of novel RSV variants raises concerns about potential changes in virulence and vaccine efficacy, emphasizing the need for continued genomic surveillance [9,10].

RSV epidemics follow a seasonal pattern, with infection peaks typically occurring during the colder months [5,6]. This seasonal increase poses additional challenges for healthcare systems, especially in regions with a high burden of chronic diseases [8,10]. While supportive care remains the primary treatment for RSV in adults, options for antiviral therapy are limited, and effective vaccines are not yet widely available for this population [9,11,12].

This study represents the first comprehensive analysis of RSV-positive adults in Croatia, conducted at the University Hospital Center Zagreb from October 2022 to April 2024. It aims to evaluate the prevalence, clinical presentations, and epidemiological characteristics of RSV in both hospitalized and outpatient populations, with a specific focus on adults aged >65 years. By identifying risk factors for severe outcomes, this study provides insights that could help guide preventive strategies, including vaccination prioritization and enhanced RSV surveillance during peak seasons.

## 2. Materials and Methods

This retrospective study was conducted at the University Hospital Center Zagreb between October 2022 and April 2024. This study analyzed the medical records of adult patients tested for respiratory infections, focusing on RSV-positive cases.

### 2.1. Study Population

This retrospective study included adult patients tested for respiratory infections at the University Hospital Center Zagreb between October 2022 and April 2024. The inclusion criteria were as follows: (1) age ≥ 18 years, (2) RT-PCR confirmation of RSV infection during the study period, and (3) availability of complete medical records.

The only exclusion criterion was the presence of incomplete medical records, which prevented a comprehensive analysis of clinical and epidemiological characteristics. Patients were categorized into three age groups: <50 years, 50–65 years, and >65 years, with a particular focus on the >65-year-old group due to their increased risk of severe outcomes and complications.

Patients with confirmed bacterial infections were not systematically excluded, as differentiation between viral and bacterial pneumonia was not always feasible due to the retrospective nature of the study. Furthermore, bacterial superinfections are a well-known complication of RSV in high-risk patients, and excluding these cases would not accurately reflect the real-world clinical burden of RSV in adults.

Similarly, patients with immunodeficiencies were not excluded, as our objective was to analyze RSV infections in a real-world hospital setting, where such high-risk patients are frequently affected. Given that immunocompromised individuals are particularly vulnerable to severe RSV outcomes, their inclusion provides valuable clinical insights into disease severity and management in this population.

Ethical approval for this study was obtained from the Ethics Committee of the University Hospital Center Zagreb (Approval Code: Klasa: 8.1-24/285-4, Broj: 02/013 AG, Date: 8 January 2025).

### 2.2. Viral Screening

Respiratory samples, including nasopharyngeal swabs, oropharyngeal swabs, and bronchoalveolar lavage (BAL) specimens, were collected following standardized hospital protocols, with sample selection based on clinical presentation and disease severity. Samples were immediately placed in viral transport media and stored at 2–8 °C for short-term analysis or at −80 °C for longer storage to ensure RNA integrity. RSV detection was conducted using reverse transcription polymerase chain reaction (RT-PCR) on two highly sensitive and specific diagnostic platforms: the Allplex™ SARS-CoV-2/FluA/FluB/RSV Assay (Seegene, Seoul, Republic of Korea) and Alinity m Resp-4-Plex Assay (Abbott, Wiesbaden, Germany). Both assays simultaneously detect RSV, SARS-CoV-2, and influenza A/B, allowing for comprehensive respiratory virus surveillance. Positive and negative controls were included in each RT-PCR run to ensure assay accuracy and rule out contamination. RNA extraction was performed using the Nucleic Acid Extraction Kit (Genrui NE48) for the Allplex™ SARS-CoV-2/FluA/FluB/RSV Assay (Seegene) and the Alinity m Sample Prep Kit 2 for the Alinity m Resp-4-Plex Assay (Abbott), following the manufacturers’ protocols. However, no further molecular characterization (e.g., genotyping) was performed as part of routine diagnostic testing. Cycle threshold (Ct) values were not included in the analysis, as the primary aim of this study was to assess the clinical and epidemiological characteristics of RSV infections rather than viral load quantification. 

### 2.3. Data Collection

Data were retrospectively collected from the Hospital Information System (HIS) and Laboratory Information System (LIS) by physicians, ensuring accuracy and consistency in data extraction. The collected variables included the following:

- Demographics: age and sex.
- Clinical presentation: categorized into upper respiratory tract symptoms (rhinorrhea, nasal congestion, and pharyngitis), lower respiratory tract symptoms (cough, dyspnea, sputum, and wheezing), general symptoms (fever, malaise, fatigue, myalgia, headache, and loss of appetite), and asymptomatic cases.
- Chronic diseases: grouped into cardiovascular diseases (e.g., heart failure and coronary artery disease), chronic respiratory conditions (e.g., COPD and asthma), diabetes mellitus, hematologic diseases, solid tumors, chronic kidney disease, liver diseases, and stroke.
- Complications: grouped into three main categories:
  1. Pneumonia: defined by clinical symptoms (fever, cough, and dyspnea), elevated inflammatory markers (C-reactive protein [CRP] and neutrophilia), and radiographic findings (consolidations or ground-glass opacities). Pneumonia included RSV-associated pneumonia and suspected bacterial pneumonia; however, a definitive distinction was not possible due to the absence of systematic microbiological sampling.
  2. Exacerbations of chronic respiratory disease: primarily COPD (chronic obstructive pulmonary disease) and asthma exacerbations.
  3. Other complications: respiratory failure, acute tracheobronchitis, sepsis, and cardiovascular or neurological disorders.
- Co-infections: Concurrent infections with influenza A/B and SARS-CoV-2 were identified using the same RT-PCR assays.
- Therapy: Data on antibiotic use and the administration of ribavirin were collected.
- Clinical outcomes: including the need for intensive care unit (ICU) admission and in-hospital mortality.

Since this was a retrospective study, some clinical variables were not systematically documented. Smoking status was not consistently recorded in medical files and was therefore excluded from the analysis, as missing data prevented a meaningful statistical evaluation. Similarly, vaccination history for RSV, influenza, and COVID-19 was incomplete, limiting the ability to assess its potential impact on RSV-related outcomes.

A proportion of asymptomatic patients were tested as part of routine hospital surveillance in high-risk departments such as hematology, oncology, and intensive care units, where early detection was crucial for preventing nosocomial transmission. Additionally, as this study was conducted during the COVID-19 pandemic, many patients underwent testing primarily to rule out SARS-CoV-2 infection, leading to the incidental detection of RSV in asymptomatic individuals due to the multiplex RT-PCR assays used. This highlights the value of broad-spectrum molecular diagnostics in identifying viral infections beyond the primary testing indication.

To ensure data reliability, all diagnoses were verified by healthcare professionals, and comorbidities were identified based on documented medical histories and prior clinical assessments recorded in the hospital database. Chronic diseases were not self-reported by patients but were extracted from medical records, including physician evaluations, laboratory findings, and imaging reports.

### 2.4. Statistical Analysis

Descriptive statistics were used to summarize patient characteristics, clinical presentation, and outcomes. Categorical variables were expressed as absolute frequencies and percentages, while continuous variables were presented as medians with interquartile ranges.

To explore associations between categorical variables, Chi-square tests were conducted. These analyses demonstrated statistically significant relationships between age and pneumonia (χ^2^ = 6.05, *p* = 0.049) and age and other complications (χ^2^ = 12.58, *p* = 0.002), while no significant difference was observed in COPD exacerbations across age groups (χ^2^ = 1.60, *p* = 0.450). Additionally, a Chi-square test comparing RSV case distributions across different seasons revealed a statistically significant difference (χ^2^ = 207.22, *p* < 0.001), indicating that RSV incidence was substantially higher during the winter months compared to other seasons.

Given the retrospective nature of this study and the limited sample size, multivariate analyses were not performed.

## 3. Results

### 3.1. RSV Prevalence and Study Population

A total of 2631 adult patients were tested for RSV during the study period, with 138 (5.25%) testing positive. The highest proportion of tested individuals belonged to the >65 years of age group (46.41%), followed by those aged <50 years (28.39%) and the 50–65 years of age group (25.20%). The RSV positivity rate was highest among individuals under 50 years (5.49%), followed by those aged ≥65 years (5.24%) and 50–65 years (4.98%).

Regarding gender distribution, males accounted for 47.10% (n = 65) and females for 52.90% (n = 73) of the RSV-positive cases. In the >65 years of age group, male and female distributions were almost equal (48.44% vs. 51.56%), whereas in the 50–65 years of age group, males were more frequently affected (54.55%). Conversely, in the <50 years of age group, females were more commonly affected (60.98%) (Table 1).

### 3.2. Seasonal Distribution of RSV Infections

The analysis of seasonal patterns revealed a clear peak in RSV cases during the winter months. From November 2022 to January 2023, cases increased significantly, with seven cases in November, twenty-two in December, and twenty-three in January. A sharp decline followed, with eight cases in February, three in March, and one in April 2023. No cases were recorded from May to October 2023. The trend resumed in December 2023, with three cases reported, followed by a marked increase in January and February 2024, each with twenty-five cases. The seasonal pattern concluded with nineteen cases in March and two cases in April 2024.

A chi-square test comparing RSV case distributions across different seasons demonstrated a statistically significant difference (χ^2^ = 207.22, *p* < 0.001), indicating that RSV incidence was substantially higher during the winter months compared to other seasons.

**Figure 1:**
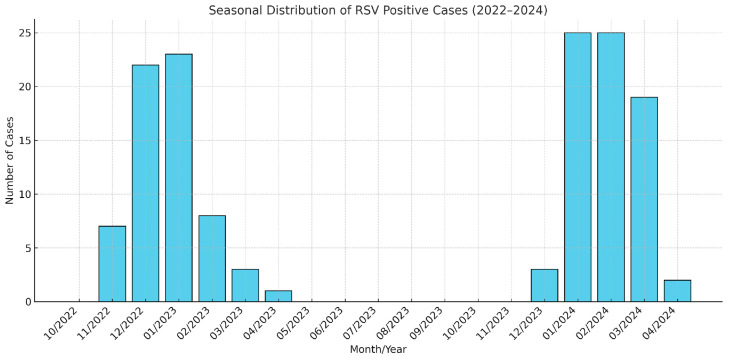
Monthly distribution of RSV-positive patients (November 2022–April 2024).

Figure 1 illustrates these findings, showing distinct peaks during winter months and negligible activity in warmer seasons. This distribution underscores the importance of allocating healthcare resources and planning preventive measures during high-incidence periods.

### 3.3. Clinical Presentation

The clinical manifestations of RSV varied in severity, with upper respiratory symptoms (rhinorrhea, nasal congestion, and pharyngitis) observed in 76.1% of patients. Lower respiratory symptoms, including cough, dyspnea, sputum production, and wheezing, were the most common, affecting 80.4% of patients. General symptoms such as fever, fatigue, and appetite loss were reported in 69.6% of patients (Table 2).

Symptom Prevalence by Age Group (Table 3):

- **Upper respiratory symptoms**—Observed across all age groups, affecting 73.1% of individuals under 50, 78.8% in the 50–65 group, and 76.6% in those over 65 years.
- **Lower respiratory symptoms**—Prevalence increased with age, ranging from 70.7% in individuals under 50 to 80.43% in those over 65 years.
- **General symptoms**—Reported in 68.3% of individuals under 50, 60.6% in the 50–65 group, and 75% in those over 65 years.
- **Asymptomatic cases**—Rare across all groups, decreasing from 9.8% in individuals under 50 to 1.6% in those over 65 years.

### 3.4. Chronic Diseases in RSV-Positive Patients

The overall prevalence of chronic diseases among RSV-positive patients was high, with 90.6% of patients having at least one chronic condition. Cardiovascular diseases were the most common chronic disease, affecting 60.9% of all patients, followed by chronic respiratory diseases (39.1%), diabetes (19.6%), and hematologic diseases and solid tumors (each at 23.2%). Chronic kidney disease affected 13.04%. Liver diseases (6.5%) and stroke (5.1%) were less prevalent (Table 4).

### 3.5. Complications in RSV-Positive Patients

Complications were common among RSV-positive patients, particularly in older adults. Pneumonia was the most frequent complication, observed in 30.4% of patients (42/138). Its prevalence increased with age: 19.5% (8/41) in patients under 50 years, 24.2% (8/33) in those aged 50–65 years, and 40.6% (26/64) in patients over 65 years. COPD or asthma exacerbations were the second most common complication, affecting 12.32% of patients (17/138). These were observed in 7.32% (3/41) of patients under 50 years, 12.12% (4/33) of those aged 50–65 years, and 15.63% (10/64) of patients over 65 years. Other complications—including respiratory failure, acute bronchitis, sepsis, cardiovascular complications, neurological disorders, and the exacerbation of pre-existing chronic diseases—were present in 17.39% of patients (24/138). Their prevalence similarly increased with age: 7.32% (3/41) in patients under 50 years, 6.06% (2/33) in the 50–65 age group, and 29.69% (19/64) in those over 65 years (Table 5).

### 3.6. Impact of Chronic Diseases on RSV Complications

Patients with chronic respiratory diseases exhibited a notably high prevalence of complications in our study. Among the 40 patients with chronic respiratory diseases, 47.5% experienced COPD exacerbations, and 45% developed pneumonia, underscoring the significant burden of respiratory complications in this group.

Other chronic conditions also contributed significantly to the incidence of pneumonia. Pneumonia was observed in 35.71% of patients with cardiovascular diseases, 44.44% of those with diabetes, 15.6% of patients with hematologic diseases, and 40.63% of patients with solid tumors. These findings highlight the role of chronic diseases in exacerbating the clinical severity of RSV infection, particularly through complications such as pneumonia. The prevalence of COPD exacerbations was highest among patients with chronic respiratory diseases (40%) but was notably lower among those with other chronic conditions: 15.5% of patients with cardiovascular diseases, 12.5% of those with solid tumors and hematologic diseases, and 11.11% of patients with diabetes. Importantly, all these patients with COPD exacerbations also had underlying chronic respiratory diseases, emphasizing the critical interplay of multimorbidity in driving COPD exacerbations within this cohort (Table 6).

### 3.7. Treatment and Clinical Outcomes

In our study, ribavirin was administered to 5.8% (8/138) of patients, predominantly within the 50–65 age group, where hematologic diseases were more prevalent (30.3%). This targeted use aligns with its established application in high-risk groups, such as immunocompromised individuals with hematologic malignancies. 

Antimicrobial therapy was prescribed to 73.91% of RSV-positive patients (102/138). The most frequently prescribed antibiotics included azithromycin (31/102), levofloxacin (26/102), and amoxicillin–clavulanic acid (21/102). Additionally, 50% of these patients received combination antibiotic therapy, with amoxicillin–clavulanic acid + azithromycin (12/51) and ceftriaxone + azithromycin (5/51) being the most commonly used regimens.

Co-infections were identified in 13.76% of patients, including COVID-19 (7.25%), influenza (4.35%), and combined COVID-19 and influenza infections (2.2%). Patients with co-infections exhibited significantly higher complication rates, with 77.78% of those co-infected with influenza and 66.7% of those with combined infections experiencing complications. 

The overall mortality rate was 2.9% (4/138), with 50% of the deaths occurring in patients co-infected with RSV and influenza. Additionally, 13.8% (19/138) of patients required admission to the intensive care unit, and an equal proportion necessitated mechanical ventilation. These results emphasize the complexity of managing RSV infections, particularly in patients with co-infections or significant comorbidities, and underscore the importance of developing targeted therapeutic strategies to improve clinical outcomes.

## 4. Discussion

This study provides new epidemiological and clinical insights into RSV infections in Croatian adults, enabling comparisons with European and North American populations.

### 4.1. Prevalence and Seasonality

The overall RSV prevalence in our study was 5.25%, which aligns with previous European studies. Korsten et al. [13] reported an RSV incidence of 4.2% during the 2017–2018 epidemic and 7.2% during the 2018–2019 epidemic among community-dwelling older adults. Their study focused solely on non-hospitalized individuals, whereas our cohort included both hospitalized and non-hospitalized patients, which may explain the comparable prevalence despite differences in study populations. The link between RSV and hospitalization has been well established. Falsey et al. [1] found RSV infection rates of 3–7% in healthy older adults and 4–10% in high-risk adults, with 16% of high-risk patients requiring hospitalization. This highlights the greater disease burden in individuals with comorbidities, reinforcing the need for targeted surveillance and preventive strategies in vulnerable populations. Seasonal analysis demonstrated a clear winter peak from November to January, consistent with previous studies in temperate regions, including Matias et al. and Shi et al., who identified similar seasonal trends in RSV activity [5,6]. These findings emphasize the importance of proactive resource allocation in high-incidence periods to optimize RSV management in high-risk adults.

### 4.2. Clinical Presentation

Our findings indicate a high prevalence of respiratory symptoms in RSV-positive adults, which aligns with previous studies. In our cohort, 76.1% of patients presented with upper respiratory tract infection (URTI), a rate that falls within the range reported by Hall [14], where 22–78% of RSV-infected adults developed signs of URTI such as nasal congestion and rhinorrhea, while sore throat was observed in 16–64% of cases. The comparability of our results with these historical data reinforces the well-established role of RSV as a significant cause of URTI in adults. Hall’s study primarily describes early-stage RSV infections, whereas our cohort includes hospitalized patients in whom disease progression is more likely. This may explain the higher prevalence of lower respiratory symptoms in our study (80.4%), reflecting a more severe clinical spectrum. This finding aligns with previous studies, where cough was reported in 85% to 95%, wheezing in 33% to 90%, and dyspnea in 51% to 93% of cases [1,2,15,16,17,18,19], indicating that lower respiratory tract involvement is a common feature of RSV infection in adults, regardless of underlying comorbidities or airway hyperreactivity. Additionally, our data support prior observations of high respiratory symptom burdens in hospitalized RSV patients. Yoon et al. [8] reported cough in 65.2%, sputum production in 68.9%, and dyspnea in 41.7% of cases, while Bouzid et al. [9] documented cough in 65% of cases, pyrexia in 45% of cases, and severe tachypnea (≥26 breaths/min). Although our study classified symptoms based on anatomical and functional groupings, leading to higher aggregated rates, the overall symptom distribution is consistent with the established clinical presentation of RSV in adults. In terms of general symptoms, we observed a prevalence of 69.6% across all patients, with rates increasing with age (68.3% in patients < 50 years, 60.6% in the 50–65 years of age group, and 75% in those over 65). This aligns with findings from Wald et al., who reported that nonspecific symptoms such as asthenia, anorexia, and fever occur in approximately 50% of cases [15]. Our higher prevalence of general symptoms in older adults suggests that RSV may present with more systemic effects in this population, potentially due to immune senescence and higher comorbidity burden. Additionally, asymptomatic RSV infection was rare in our cohort, observed in only 5.1% of cases overall, with the prevalence decreasing with age (9.8% in individuals < 50 years, 1.6% in those ≥ 65 years). This finding is consistent with previous reports indicating that RSV infection in adults, particularly in older populations, is typically symptomatic. Falsey et al. found that asymptomatic RSV infection occurs in fewer than 5% of adults, reinforcing the notion that while subclinical cases are possible, they are uncommon [20,21].

These findings highlight the broad symptomatic spectrum of RSV in adults, ranging from mild upper respiratory symptoms in outpatients to more severe lower respiratory tract involvement requiring hospitalization. The variation in disease severity is further emphasized by Korsten et al. [13], who found no severe RSV cases or hospitalizations in a European community-dwelling cohort, suggesting that hospital-based studies capture a more severe disease burden, while community studies predominantly report milder cases. This discrepancy underscores the need for more comprehensive RSV surveillance, particularly in older adults with comorbidities, where disease severity may be underestimated in non-hospitalized populations.

### 4.3. Chronic Diseases and Complications

Our study demonstrates a high prevalence of chronic diseases among RSV-positive adults in Croatia, with 90.6% having at least one chronic condition. Cardiovascular diseases (60.9%) were the most common comorbidity, followed by chronic respiratory diseases (39.1%), diabetes (19.6%), and hematologic malignancies and solid tumors (both 23.2%), findings consistent with previous studies in hospitalized RSV patients (Widmer et al., Branche et al.) [2,17].

A comparison with the Italian study [22], which focused on older adults in primary care, and the Danish study [11], which analyzed hospitalized RSV patients, reveals both similarities and notable differences in the distribution of chronic diseases and complications. In both the Croatian and Italian studies, cardiovascular diseases were the most prevalent comorbidity, affecting 69.7% of RSV-positive patients in Italy and 60.9% in our cohort. However, chronic respiratory diseases were more common in our study (39.1%) compared to 24.2% in the Italian study, likely reflecting the higher proportion of hospitalized patients in our cohort, as individuals with pre-existing lung conditions are at a greater risk of severe RSV infections requiring inpatient care. The prevalence of diabetes was also slightly higher in our study (19.6% vs. 15.1%).

Pneumonia was a frequent complication, occurring in 30.4% of RSV-positive adults, with higher rates among older adults (40.6% in those ≥ 65 years) and those with chronic respiratory diseases (45%). These findings align with those of the Danish study [11], which reported pneumonia in 41% of hospitalized RSV patients, a rate significantly higher than that observed in influenza A (29%) and influenza B (24%). The higher pneumonia rates in hospitalized cohorts (Danish study and our study) suggest that RSV may lead to more severe lower respiratory tract infections in high-risk patients compared to those seen in primary care settings (Italian study) [11,22].

Exacerbations of chronic respiratory diseases (COPD and asthma) were observed in 12.3% of our patients, a complication not explicitly addressed in the Italian study but well documented in previous hospital-based studies (Widmer et al., Bouzid et al.) [2,9]. Among chronic disease subgroups, patients with chronic respiratory diseases exhibited the highest rates of pneumonia (45%) and COPD exacerbations (47.5%), underscoring the significant burden of RSV complications in this group. Pneumonia was also prevalent in patients with diabetes (44.4%), solid tumors (40.6%), cardiovascular diseases (35.7%), and hematologic diseases (15.6%). In addition, 15.6% of older adults (>65 years) experienced COPD exacerbations, further confirming that RSV frequently leads to acute respiratory decompensation in individuals with underlying pulmonary conditions (Falsey et al., Lee et al.) [1,12].

Beyond respiratory complications, RSV has been linked to acute cardiac events, including congestive heart failure (CHF), arrhythmias, acute coronary syndromes, and myocarditis [1,23]. Additionally, bacterial and viral co-infections significantly worsen disease severity and mortality. In hospitalized adults with RSV-associated lower respiratory tract infections (LRTIs), bacterial co-infection rates range from 12.5% to 23.4%, while viral co-infections are observed in 21.8% of cases [12,24].

In immunocompromised individuals, RSV can rapidly progress from upper to lower respiratory tract disease in 40–60% of cases, with mortality rates reaching up to 80% in hematopoietic stem cell transplant (HCT) recipients [25,26,27,28,29]. In our study, ribavirin was administered in 5.8% of cases, predominantly in immunocompromised patients, reflecting current clinical practices prioritizing antiviral therapy for high-risk groups.

These findings highlight the complexity of the patient population managed at University Hospital Center Zagreb, a tertiary care center specializing in advanced chronic conditions and severe disease presentations. The high prevalence of complications among older adults and multimorbid patients emphasizes the clinical burden of RSV in these groups. Compared to international studies, our findings align with research from the United States and Europe, where RSV is increasingly recognized as a major cause of hospitalization in older adults with chronic diseases (Widmer et al., Falsey et al., Bouzid et al.) [1,2,9,20]. However, specific public health challenges in Croatia—such as high smoking rates and the burden of cardiovascular disease—may contribute to the distinct clinical profile observed in our cohort. Addressing these risk factors, improving RSV surveillance, and optimizing management strategies may help reduce severe RSV outcomes in high-risk populations.

### 4.4. Treatment and Prevention

Antimicrobial therapy was prescribed to 73.9% of our patients, a notably higher proportion than in the Italian study (59.2%) [22]. This difference likely reflects variations in clinical management between inpatient and outpatient settings, with hospitalized patients being more likely to receive empiric antibiotic treatment due to concerns about bacterial superinfection. However, despite the widespread use of antibiotics, the microbiological confirmation of bacterial infections was not systematically performed, making it difficult to distinguish between viral and bacterial etiologies.

Similar trends have been observed in previous studies, where antibiotics were frequently prescribed despite the absence of confirmed bacterial co-infections (Falsey et al., Widmer et al., Yoon et al.) [1,2,8]. The frequent use of antibiotics in our cohort likely reflects clinical decision-making aimed at covering potential bacterial pathogens in the absence of definitive microbiological evidence. In many cases, antibiotic therapy was initiated empirically based on clinical symptoms, laboratory tests, and radiographic findings rather than confirmed bacterial infections. However, microbiological cultures were not systematically performed, limiting our ability to assess the true burden of bacterial co-infections in RSV patients.

These findings underscore the need for improved antimicrobial stewardship programs in RSV management, particularly in hospital settings where empiric antibiotic therapy remains common. Future studies incorporating comprehensive microbiological testing could provide better insights into the appropriateness of antibiotic use in RSV-infected patients, potentially helping to reduce unnecessary antibiotic exposure and associated risks, such as antimicrobial resistance.

Currently, RSV treatment remains primarily supportive, with ribavirin being the only approved antiviral therapy for RSV disease [10]. However, its use is limited to select high-risk patients, particularly immunocompromised individuals, due to concerns about its efficacy in mild cases and potential adverse effects [9]. In our study, ribavirin was administered to 5.8% of patients, predominantly in the 50–65 age group, where hematologic diseases were most prevalent (30.3%). This selective use reflects current clinical practice, prioritizing antiviral therapy for patients at the highest risk of severe complications [9,10].

The recent approval of two RSV vaccines for older adults (Arexvy–GSK and Abrysvo–Pfizer) and a long-acting monoclonal antibody (Nirsevimab) for infant protection represents a major advancement in RSV prevention [30]. Future studies will need to evaluate the duration of protection, the optimal timing of vaccination, and the safety of co-administration with other seasonal vaccines, such as influenza and SARS-CoV-2 [10]. Additionally, while Nirsevimab is currently indicated for infants, its potential use in high-risk adults, particularly immunocompromised patients with reduced vaccine efficacy, warrants further investigation [30].

Antiviral treatment options for RSV remain limited, but new oral and inhaled antivirals are in development [31]. However, their efficacy in clinical settings has been inconsistent, potentially due to differences in immune profiles and the timing of drug administration, which is a known challenge in respiratory viral infections [32]. Given the potential for RSV genetic evolution, continuous surveillance is essential to monitor viral variants and their impact on vaccine and monoclonal antibody efficacy, ensuring the long-term effectiveness of preventive and therapeutic strategies [30,33].

### 4.5. Co-Infections

Co-infections were identified in 13.76% of patients, including SARS-CoV-2 (7.25%), influenza (4.35%), and concurrent SARS-CoV-2 and influenza infections (2.2%). Patients with co-infections exhibited significantly higher complication rates, with 77.78% of those co-infected with influenza and 66.7% of those with both SARS-CoV-2 and influenza experiencing complications. These findings align with Havers et al., where viral co-infections were identified in 4.6% of hospitalized RSV cases, although no significant increase in severity was observed in their cohort [34]. Our results are also consistent with previous reports by Yoon et al. and Bouzid et al., which emphasize the role of co-infections in exacerbating complications, such as pneumonia, and increasing the need for ICU admission [8,9]. The higher complication rate among co-infected patients in our study suggests that RSV, in combination with influenza or SARS-CoV-2, may contribute to a more severe clinical course. These findings highlight the need for enhanced surveillance and preventive strategies in high-risk populations.

### 4.6. Severe Outcomes and Mortality

ICU admission was required in 13.8% of our patients, which is lower than the 17.0% observed by Havers et al. and the 26.9% reported by Schmidt et al. However, the need for mechanical ventilation in our study (13.8%) was comparable to that reported by Schmidt et al. (12.3%) but higher than the 4.8% observed by Havers et al. [34,35]. These discrepancies may be attributed to differences in patient selection criteria, hospital admission thresholds, and regional variations in clinical management. Our findings align with previous systematic reviews on RSV epidemiology, which report ICU admission rates ranging from 10% to 31% and mechanical ventilation requirements between 3% and 17% among hospitalized adults with an RSV infection [36].

Our findings further highlight the severity of RSV in older adults with comorbidities. Similarly to the study by Havers et al., COPD and congestive heart failure were significantly associated with severe outcomes [34]. Given that these conditions have also been identified as key risk factors in previous studies, our results reinforce the importance of targeted preventive measures, including RSV vaccination, for high-risk populations [10].

The overall mortality rate in our study was 2.9%, with 50% of deaths occurring in patients co-infected with RSV and influenza. This rate is lower than the 4.7% reported by Havers et al. [34] and the 3.9% reported by Schmidt et al. [35], who conducted a six-year retrospective study on hospitalized RSV patients. Differences in study populations likely account for these variations; the study by Havers et al. [34] included a higher proportion of long-term care facility residents (17.2%), whereas our cohort comprised predominantly community-dwelling patients requiring hospitalization.

## 5. Limitations

This study provides valuable insights into the epidemiology and clinical characteristics of RSV in adults; however, several limitations should be considered when interpreting the results.

As a retrospective study, the findings are dependent on the accuracy and completeness of medical records, which may introduce selection and information bias. Additionally, the absence of a control group of RSV-negative patients limits the ability to precisely determine the impact of RSV infection compared to other respiratory viruses or non-viral respiratory conditions.

Another limitation is the lack of information on the vaccination status against respiratory viruses such as RSV, influenza, and SARS-CoV-2. Given the potential protective effects of vaccination, these missing data prevent a more comprehensive assessment of how immunization status may have influenced disease severity and outcomes.

Microbiological analyses to differentiate between bacterial and viral pneumonias were not systematically performed. While pneumonia was a frequent complication, the inability to distinguish between RSV-associated and bacterial pneumonias limits conclusions regarding the role of secondary bacterial infections. This also affects the evaluation of antimicrobial prescribing practices in RSV patients.

Additionally, RSV genotyping was not performed, which precluded the identification of circulating viral strains and potential genomic variations. While genotypic surveillance would provide valuable epidemiological data, obtaining genetic sequencing for all RSV-positive patients was not feasible within the scope of this study.

Lastly, statistical significance tests were not conducted for all comparisons due to sample size limitations, and future studies with larger cohorts should incorporate multivariable analyses to better define independent risk factors for severe RSV outcomes.

Despite these limitations, this study offers important epidemiological and clinical insights into RSV in adults, particularly in a tertiary care setting. The findings emphasize the burden of RSV among older adults and those with chronic conditions, supporting the need for enhanced surveillance, preventive strategies, and targeted clinical management.

## 6. Conclusions

This study represents the first comprehensive analysis of RSV infections in Croatian adults, highlighting key epidemiological trends, clinical characteristics, and disease burden. The overall prevalence of RSV was 5.25%, consistent with findings from other European studies. The infection followed a seasonal pattern, with a clear peak during the winter months (December–February), reinforcing the need for enhanced surveillance and resource allocation during high-incidence periods.

Older adults (≥65 years) and individuals with chronic diseases bore the greatest burden of severe outcomes, including pneumonia (40.63%), COPD exacerbations (15.63%), ICU admissions (13.8%), and mortality (2.9%). The high prevalence of chronic cardiovascular (60.9%) and respiratory diseases (39.1%) in the Croatian RSV-positive population was similar to findings in Western Europe but higher than in some other regions, suggesting potential differences in population health status and healthcare utilization. Compared to international data, our study showed a higher frequency of antibiotic use (73.91%), often without microbiological confirmation, highlighting the need for improved antimicrobial stewardship and RSV-specific diagnostic algorithms.

RSV co-infections with influenza and SARS-CoV-2 were associated with worse clinical outcomes, particularly in patients with multiple comorbidities. While ribavirin was used in a limited number of high-risk patients (5.8%), no other targeted antiviral treatments were available, underscoring the current lack of effective RSV-specific therapies for adults.

The recent approval of RSV vaccines for older adults represents a major advancement in prevention. However, further studies are needed to evaluate their long-term efficacy, optimal vaccination strategies, and co-administration with seasonal influenza and COVID-19 vaccines. Additionally, improved RSV surveillance and the genotypic analysis of circulating strains will be essential to assess potential viral evolution and resistance patterns.

In conclusion, our findings emphasize the substantial disease burden of RSV in older adults and high-risk populations, highlighting the need for improved diagnostic, therapeutic, and preventive strategies. Targeted vaccination programs, better clinical awareness, and enhanced surveillance will be crucial in reducing RSV-related morbidity and mortality in Croatia and beyond. Future research should focus on prospective studies, the genomic characterization of RSV strains, and evaluating the real-world impact of vaccination to guide evidence-based public health interventions.

## Figures and Tables

**Table 1:** Demographic and RSV positivity rates by age group and gender.

Age Group	Total Tested (%)	RSV Positive (%)	Males (n, %)	Females (n, %)
<50 years	747 (28.39%)	41 (5.49%)	16 (39.02%)	25 (60. 98%)
50–65 years	663 (25.20%)	33 (4.98%)	18 (54.55%)	15 (45.45%)
>65 years	1221 (46.41%)	64 (5.24%)	31 (48.44%)	33 (51.56%)
Total	2631 (100%)	138 (5.25%)	65 (47.10%)	73 (52.90%)

**Table 2:** Clinical presentation of RSV-positive patients.

Symptom Type	Number of Patients (n)	Percentage (%)
Upper respiratory symptoms	105	76.09
Lower respiratory symptoms	111	80.43
General symptoms	96	69.57
Asymptomatic cases	7	5.07

**Table 3:** Symptom prevalence by age group.

Symptom Type	Overall (n = 138)	<50 years (n = 41)	50–65 years (n = 33)	>65 years (n = 64)
Upper respiratory symptoms	76.09% (105/138)	73.17% (30/41)	78.79% (26/33)	76.56% (49/64)
Lower respiratory symptoms	80.43% (111/138)	70.73% (29/41)	78.79% (26/33)	87.5% (56/64)
General symptoms	69.57% (96/138)	68.29% (28/41)	60. 61% (20/33)	75% (48/64)
Asymptomatic cases	5.07% (7/138)	9.76% (4/41)	6.10% (2/33)	1.56% (1/64)

**Table 4:** Prevalence of chronic diseases and comorbidities by age group.

Chronic Disease	Overall (n = 138)	<50 Years (n = 41)	50–65 Years (n = 33)	>65 Years (n = 64)
Cardiovascular diseases	60.87% (84/138)	24.39% (10/41)	48.48% (16/33)	90.63% (58/64)
Chronic respiratory diseases (COPD, asthma)	28.99% (40/138)	14.63% (6/41)	27.27% (9/33)	39.06% (25/64)
Diabetes	19.56% (27/138)	7.32% (3/41)	18.18% (6/33)	28.13% (18/64)
Hematologic diseases	23.19% (32/138)	24.39% (10/41)	30.30% (10/33)	18.75% (12/64)
Solid tumors	23.19% (32/138)	12.20% (5/41)	21.21% (7/33)	31.25% (20/64)
Chronic kidney disease	13.04% (18/138)	9.76% (4/41)	15.15% (5/33)	14.06% (9/64)
Liver diseases	6.52% (9/138)	4.89% (2/41)	15.15% (5/33)	3.13% (2/64)
Stroke	5.07% (7/138)	0.00% (0/41)	0.00% (0/33)	10.94% (7/64)

**Abbreviations**: COPD—chronic obstructive pulmonary disease.

**Table 5:** Complication rates by age group.

Complication	Overall (n = 138)	<50 Years (n = 41)	50–65 Years (n = 33)	>65 Years (n = 64)
Pneumonia	30.43% (42/138)	19.51% (8/41)	24.24% (8/33)	40.63% (26/64)
Exacerbations of chronic respiratory disease	12.32% (17/138)	7.32% (3/41)	12.12% (4/33)	15.63% (10/64)
Other complications	17.39% (24/138)	7.32% (3/41)	6.06% (2/33)	29.69% (19/64)

**Table 6:** Association between comorbidities and complications in RSV-positive patients.

Chronic Disease	Total Patients	Pneumonia (%)	COPD Exacerbation (%)
Chronic respiratory diseases	40	45% (18/40)	47.5% (19/40)
Cardiovascular diseases	84	35.71% (30/84)	15.48% (13/84)
Diabetes	27	44.44% (12/27)	11.11% (3/27)
Hematologic diseases	32	15.6% (5/32)	12.5% (4/32)
Solid tumors	32	40.63% (13/32)	12.5% (4/32)

## Data Availability

Data supporting the findings of this study are available from the corresponding author upon reasonable request. Due to privacy regulations, patient-level data cannot be publicly shared.
